# DNA‐damage RBE assessment for combined boron and gadolinium neutron capture therapy

**DOI:** 10.1002/acm2.14399

**Published:** 2024-05-20

**Authors:** Reza Shamsabadi, Hamid Reza Baghani

**Affiliations:** ^1^ Physics Department Hakim Sabzevari University Sabzevar Iran

**Keywords:** boron‐10, gadulunium‐157, Monte Carlo simulation, neutron capture therapy, relative biological effectiveness

## Abstract

**Purpose:**

Neutron capture therapy (NCT) by 10B and 157Gd agents is a unique irradiation‐based method which can be used to treat brain tumors. Current study aims to quantitatively evaluate the relative biological effectiveness (RBE) and dose distributions during the combined BNCT and GdNCT modalities through a hybrid Monte Carlo (MC) simulation approach.

**Methods:**

Snyder head phantom as well as a cubic hypothetical tumor was at first modeled by Geant4 MC Code. Then, the energy spectra and dose distribution relevant to the released secondary particles during the combined Gd/BNCT were scored for different concentrations of 157Gd and 10B inside tumor volume. Finally, the scored energy spectra were imported to the MCDS code to estimate both RBESSB and RBEDSB values for different 157Gd concentrations.

**Results:**

The results showed that combined Gd/BNCT increases the fluence‐averaged RBESSB values by about 1.7 times when 157Gd concentration increments from 0 to 2000 µg/g for both considered cell oxygen levels (pO_2_ = 10% and 100%). Besides, a reduction of about 26% was found for fluence‐averaged RBEDSB values with an increment of 157Gd concentration in tumor volume.

**Conclusion:**

From the results, it can be concluded that combined Gd/BNCT technique can improve tumor coverage with higher dose levels but in the expense of RBEDSB reduction which can affect the clinical efficacy of the NCT technique.

## 1 INTRODUCTION

Glioblastoma is one of the most common primary malignant brain tumor among adults with an approximate incidence rate of 5 to 10 persons per 100 000 populations.^1^, ^2^, ^3^ Tumor resection along with radiotherapy and adjuvant chemotherapy techniques result in a mean survival of about 14 months after glioblastoma treatment.^4^ Recently, neutron capture therapy (NCT) as a bimodal radiotherapy technique using boron and gadolinium (known as the BNCT and GdNCT modalities, respectively) appears to be a promising modalities for the treatment of brain malignancies.^5^, ^6^, ^7^

NCT by the ^10^B agents has a large thermal neutron capture cross‐section of 3840 barns.^8^, ^9^ In this technique ^10^B can be selectively concentrated within the tumor cells and tumor boundary would be precisely determined.^10^ Neutron capture process by ^10^B results in nuclear reaction of ^10^B (n_th_, α) ^7^Li which is shown in following equation^11^:

(1)

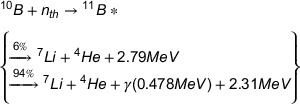




During the neutron capture process by Boron agents, released high‐LET (linear energy transfer) particles have a short range in tissues (about 9 µm for ^4^He particles and 5 µm for the ^7^Li) compared with the cellular dimensions. Therefore, selective distribution of the Boron agents inside the tumor region (known as the unique aspect of BNCT) causes a high deposited dose in tumor cells.^12^, ^13^ Accordingly, high tumor cells killing efficiency can be expected for brain tumor treatment through NCT with ^10^B agent.^8^, ^14^ To deliver the therapeutic dose during the BNCT technique, a uniform concentration of boron agents per gram tumor should be approximately about 10 to 30 µg.^15^ Due to the high LET values of the released secondary particles during the BNCT (^4^He and ^7^Li), only nearby cells to the reaction point are damaged without affecting the adjacent healthy tissues.^16^

Although secondary particles with lower LET values (electrons and photons) are released during the GdNCT, ^157^Gd has a higher neutron capture cross‐section with respect to ^10^B which can lead to lower neutron flux required for NCT technique. Besides, these released secondary particles after neutron capture by ^157^Gd have long ranges inside the target volume. Hence, this issue may result in improving the dose uniformity inside the treatment target when NCT technique is used for tumor irradiation.

Totally, GdNCT releases low‐LET prompt gamma rays, internal conversion (IC) electrons, x‐rays, and Auger electrons which are shown in following equitation^17^, ^18^:

(2)

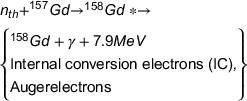




In each neutron capture reaction by ^157^Gd, the mean energy of released gamma rays is about 2.4 MeV which can penetrate to much larger depths than the Boron reaction products. Produced Auger electrons with the average LET of 300 keV/µm can result in a large probability of producing lethal damage especially when directly bound to the DNA molecule.^19^ To obtain proper results during the tumor treatment via GdNCT technique, the ^157^Gd concentration of about 50 to 200 µg/g tumor has been reported. Nevertheless, for deep tumors more ^157^Gd concentrations may be required because the incident neutron can be remarkably attenuated by overlying tissues. Therefore, higher ^157^Gd concentrations are used to ensure that required neutron capture interactions are occurred inside the tumor volume.^19^, ^20^ Considering the energy of released gamma rays and electrons during the neutron capture by ^157^Gd, the released gamma rays and Auger electrons have the range of some centimeter (cm) and a few micrometers (µm) in tissue, respectively.^21^, ^22^

Owing to the different released secondary particles during the NCT by boron and gadolinium, the therapeutic effects of GdNCT and BNCT can be quite different. The biological effectiveness of BNCT depends on the short range ^4^He and ^7^Li particles, while GdNCT techniques rely on emitted Auger electrons.^23^ Furthermore, as mentioned previously, BCNT and GdNCT have their own merits and limitations. Higher levels of DNA damage by released secondary high LET particles during the BNCT, while improved dose uniformity would be expected for GdNCT technique with high range secondary particles. Therefore, it may be expected that a combination of a ^10^B and ^157^Gd may influence the treatment outcomes in terms of dose uniformity and DNA‐damage RBE. Despite the ^157^Gd advantages, only a few studies have been assessed the combinations of BNCT and GdNCT which relevant in vivo studies of such combination are still rare.^10^ To the best of our knowledge, the radiobiological characteristics of combined ^10^B/^157^Gd NCT have not been assessed. The present study aims to evaluate the potential clinical improvement of a combined ^10^B/^157^Gd NCT technique viewpoint the dose distribution and DNA‐damage relative biological effectiveness (RBE) values through a hybrid Monte Carlo (MC) simulation approach.

## 2 METHODS

### 2.1 MC simulation

To calculate the dose distribution and DNA‐damage RBE values relevant to the combined BNCT and GdNCT techniques, at first the proposed head phantom by Snyder et al.,^24^ was simulated with Geant4.11.0 MC Code. To do so, different parts of the employed head phantom including skin, skull, and brain were simulated using the following ellipsoidal equations:

(3) $${\left(\frac{x}{6}\right)}^{2}+{\left(\frac{y}{9}\right)}^{2}+{\left(\frac{z-1}{6.5}\right)}^{2}=1\begin{array}{c} \mathrm{brain} \end{array}$$

(4) $${\left(\frac{x}{6.8}\right)}^{2}+{\left(\frac{y}{9.8}\right)}^{2}+{\left(\frac{z}{8.3}\right)}^{2}=1\mathrm{skull}$$

(5) $${\left(\frac{x}{7.3}\right)}^{2}+{\left(\frac{y}{10.3}\right)}^{2}+{\left(\frac{z-1}{8.8}\right)}^{2}=1\mathrm{skin}$$

It should be mentioned that numbers and parameters in these equations are in terms of cm.

The elemental compositions of the simulated regions relevant to the Snyder phantom were taken from ICRU‐46 report and have been listed in Table 1.^25^

**TABLE 1:** The elemental compositions relevant to the different simulated parts of employed head phantom by weight fraction.^25^

	Elemental weight fractions (%)											
	H	O	C	Na	Ca	K	Cl	P	S	N	Mg	Density (g/cm^3^)
**Brain**	10.7	71.2	14.5	0.2	0	0.3	0.3	0.4	0.2	2.2	0	1.04
**Skull**	5.0	43.5	21.2	0.1	17.6	0	0	8.1	0.3	4.0	0.2	1.61
**Skin**	10.0	64.5	20.4	0.2	0	0.1	0.3	0.1	0.2	4.2	0	1.09

**FIGURE 1:**
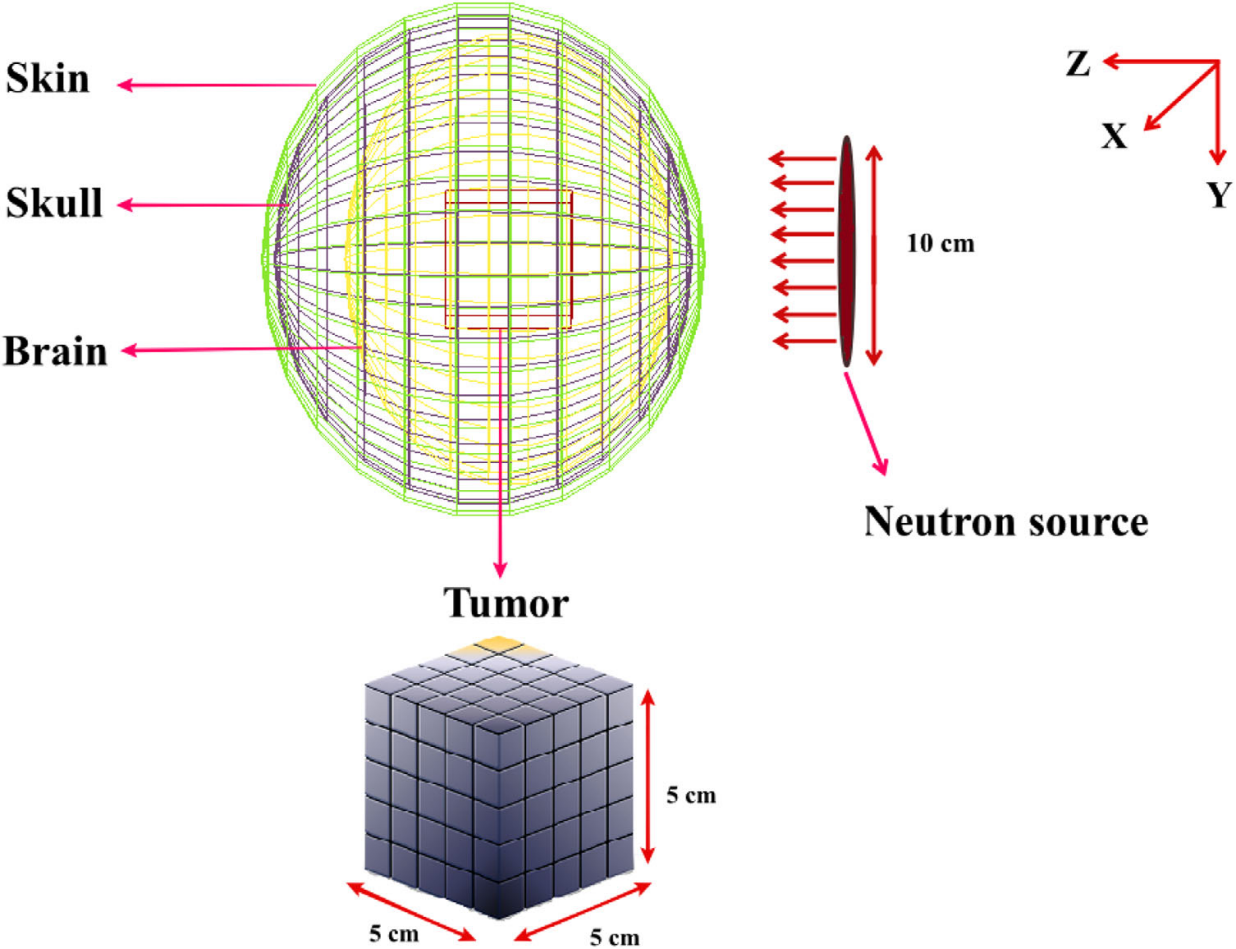
Simulated Snyder head phantom and a cubic brain tumor through Geant4 MC Code.

Furthermore, a hypothetical cubic brain tumor with the dimensions of 5 × 5 × 5 cm^3^ was considered at the center of brain volume to score the released secondary particles energy spectra as well as dose distribution calculation. The Geant4‐based simulated MC model of the Snyder head phantom along with the considered phantom inside the brain are illustrated in Figure 1.

For the tumor irradiation, the neutron source was similar to the proposed one for BNCT of brain tumors.^26^ In this regard, a disk shape source with 10 cm diameter was considered which had been placed 15 cm away from the brain center along the z‐axis. Totally, the neutron source was included 89% of epithermal neutron beams (in the range of 0.5 eV to 10 keV to increase the neutron beam penetration depth) along with 1% fast neutrons contamination (from 10 keV to 2 MeV to increment the neutron beam penetrations through the skull) and 10% thermal neutrons (form 0.001 to 0.5 eV).^26^

**FIGURE 2:**
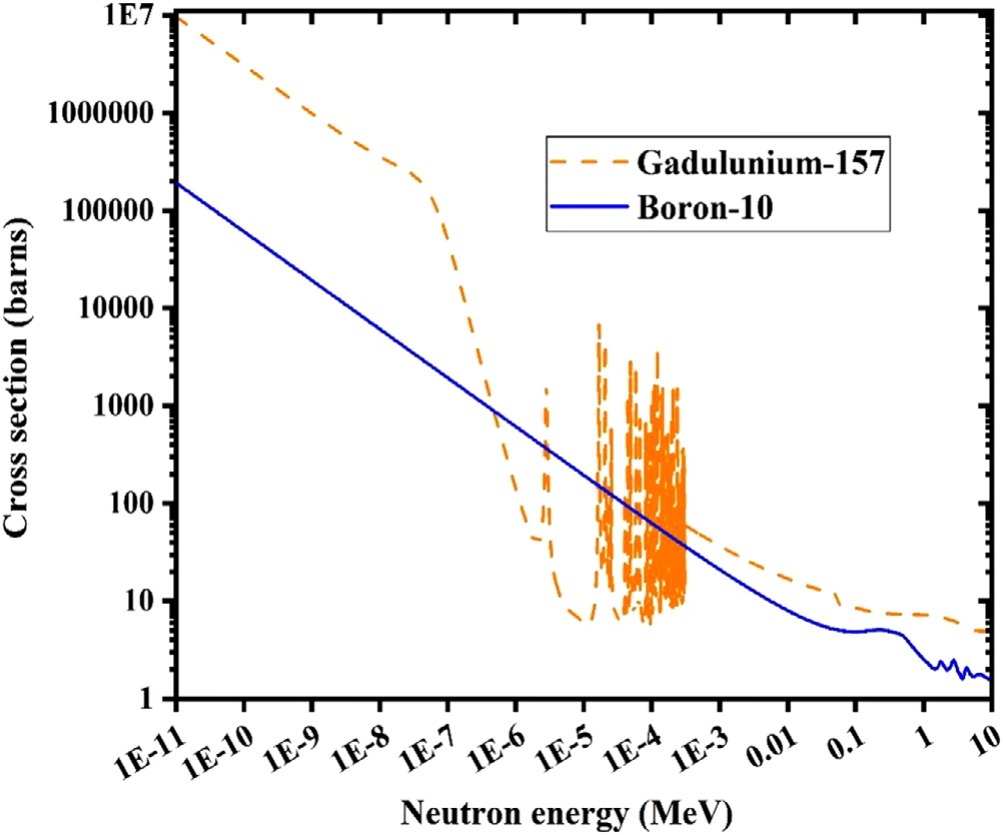
Comparison of the total neutron cross‐section relevant to ^157^Gd and ^10^B.^27^

Employed neutron cross‐sections of ^10^B and ^157^Gd have been depicted in Figure 2.

It should be mentioned that simulations of the secondary particles were performed by considering 30 µg/g ^10^B concentration and different ^157^Gd concentrations of 0, 125, 250, 500, 1000, and 2000 µg/g within the simulated tumor, as suggested by Culbertson and Jevremovic study.^15^ Since Boron compounds may be also accumulated in the healthy brain cells, a 10 µg/g tumor of ^10^B concentration was also simulated inside the surrounding normal brain region. It is worth to note that for energy spectrum and dose calculations, a uniform distribution of boron and gadolinium was considered inside the tumor volume.

The G4HadronPhysicsQGSP_BERT_HP, G4RadioactiveDecayPhysics, and G4EmStandardPhysics‐option4 physics were employed to model the neutron capture and electromagnetic process during the tumor irradiation with neutron source. To reduce the statistical errors below 1%, one billion primary particles were tracked by an Intel Core i7/8 GB RAM personal computer. Besides, the range cut‐off value in all simulations was set to 0.1 µm to score all released electrons, particles, and ions.

### 2.2 Dose calculations

To evaluate the variations of dose distribution within the tumor volume with changing the ^157^Gd concentrations during the combined Gd/BNCT technique, the tumor volume was divided into cubic voxels with the dimensions of 1 × 1 × 1 mm^3^ and absorbed dose were scored in each voxel. Then, the dose distributions relevant to each considered ^157^Gd concentration within the tumor volume were reported.

### 2.3 RBE estimations

To calculate the RBE values related to the combined of Gd/BNCT technique, the last released version of Monte Carlo damage simulation (MCDS 3.10A) code was utilized. In this regard, at first secondary particles energy spectra during the combined Gd/BNCT with different ^157^Gd concentrations were calculated inside the tumor volume. Then, the obtained data were separately imported to the MCDS MC code to estimate the relevant fluence‐averaged DNA damage yields for each type of released secondary particle. The MCDS output file is the strand break yields (single strand and double strand breaks) per Gray per Giga base pair (in terms of Gy^−1^ Gbp^−1^). Corresponding RBE values of released secondary particles (secondary electrons, alpha, and lithium) during the combined B/GdNCT technique for different concentrations of the ^10^B and ^157^Gd were separately estimated by dividing the calculated break yields to the obtained break yields for ^60^Co gamma rays as a reference radiation. It is worth mentioning that the double strand break (DSB) and single strand break (SSB) yields for ^60^Co reference radiation were considered as 8.32 and 188.84 Gy^−1^ Gbp^−1^, respectively.^28^, ^29^ Finally, to evaluate the average levels of DNA damage at a multi‐cellular scale during the combined B/GdNCT technique, the fluence‐averaged RBE was determined by considering the effects of absorbed dose to the target volume through Equation (6)^30^, ^31^:

(6) $$RBE=\frac{\sum_{i}\left(RBE_{i}\times D_{i}\right)}{\sum_{i}D_{i}}$$

*D*_i_ indicates the received dose (in Gy) by the brain tumor from i^th^ particle type which has been simulated by Geant4 MC code and RBE_i_ represents the relevant fluence‐averaged RBE value of the i^th^‐type charged particle resulted from MCDS MC code.

Validity of MCDS for DNA damage calculations has been confirmed by Semenenko and Stewart.^32^ The MCDS algorithm for DNA damage classification differs from Monte Carlo track structure (MCTS) codes. Nevertheless, a proper agreement between the results of MCDS and MCTS codes has been reported.^33^, ^34^, ^35^ On other words, MCDS parameters for DNA damage calculation including minimum length of undamaged base pairs between neighboring elementary damages (*n_min_)*, the ratio of base damage to strand break (*f*), the number of individual strand breaks (*σ_Sb_)*, and DNA segment length in terms of base pairs (*n_seg_*) have been properly tuned to have the minimum difference compared with MCTS codes.^36^ This algorithm allows to simulate the DNA damage in irradiated cells with photons, mono‐energetic electrons, protons, and particles up to ^56^Fe with the maximum energy of 1 GeV.^36^ If the induced lesions over one or two strands of the DNA molecule do not face with the 10 bp (base pair) it would be marked as the SSB breaks. Besides, two single strand breaks found on the opposite DNA strand within 10 bp are marked as the DSB. The remaining ones are classified as base damaged (BD) damage.^32^, ^37^

MCDS code can predict the type of DNA lesions which formed by ionizing radiation in typical mammalian cells under different cell oxygenation levels through considering the free radicals which are produced during the interaction of ionizing radiation with cell cytoplasm. It is worth mentioning that the strand breaks yield calculations (both SSB and DSB yields) in present study were performed in fully aerobic (pO_2 _= 100%) and hypoxia (pO_2 _= 10%) conditions to evaluate the impact of cell oxygenation level on the RBE values as well. Furthermore, all MCDS simulations have been performed with a mean standard error of less than 0.1%.

## 3 RESULTS AND DISCUSSION

### 3.1 Secondary charged particles energy spectra

**FIGURE 3:**
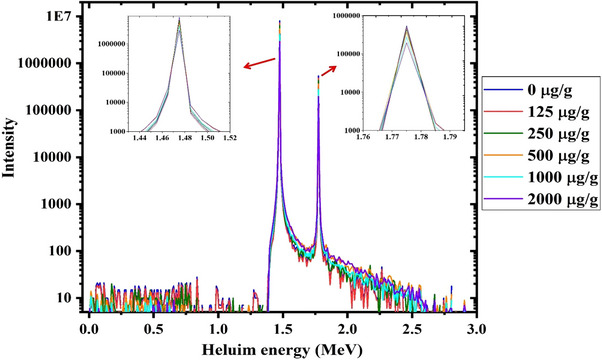
Scored energy spectra of released ^4^He particles during the neutron capture process by ^10^B at different ^157^Gd concentrations during the combined Gd/BNCT technique.

The scored ^4^He energy spectra relevant to the 30 µg/g of ^10^B inside the tumor volume in combination with different ^157^Gd concentrations (i.e., 0, 125, 250, 500, 1000, and 2000 µg/g tumor) have been illustrated in Figure 3.

As depicted in Figure 3, the intensities of scored ^4^He energy spectra during the neutron capture process by ^10^B decrement by increasing the ^157^Gd concentrations from 0 to 2000 µg/g tumor. With increasing the gadolinium concentration, boron neutron capture reactions decrement due to the high thermal neutron cross‐section of gadolinium agents. Hence, the released secondary particles through the ^10^B neutron capture process decrease. This reduction in intensity of helium energy spectra during the combined Gd/BNCT technique may influence the RBE value.

**FIGURE 4:**
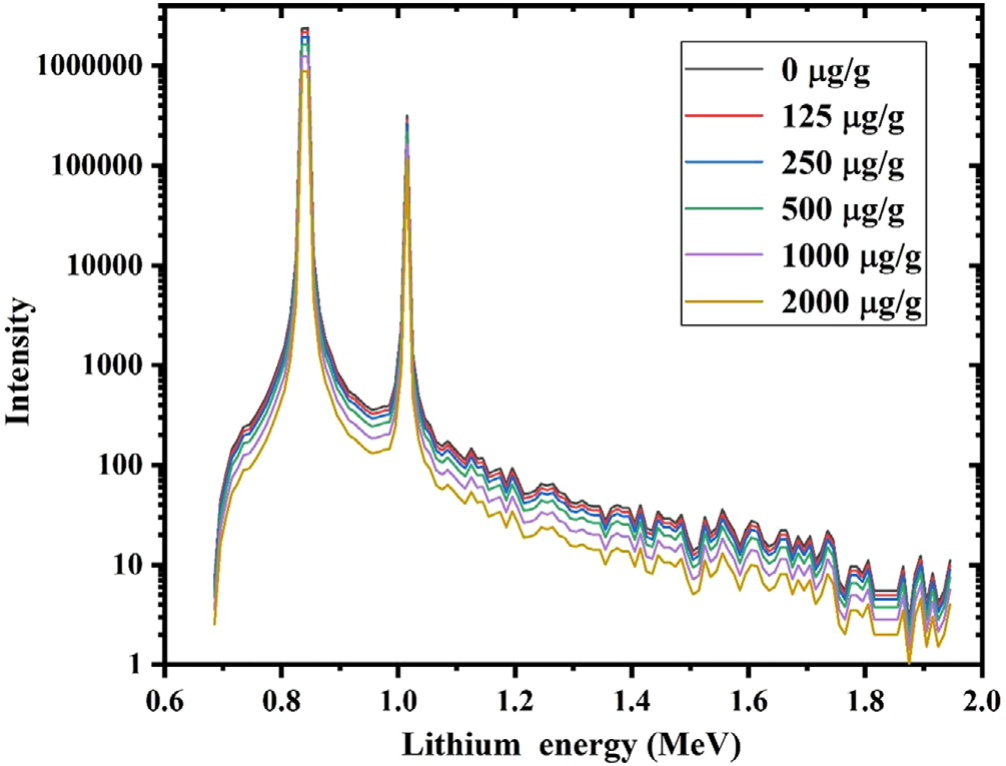
Scored energy spectra of released ^7^Li particles during the neutron capture process by ^10^B at different ^157^Gd concentrations during Gd/BNCT combination.

The obtained ^7^Li energy spectra relevant to 30 µg/g ^10^B and different ^157^Gd concentrations (i.e., 0, 125, 250, 500, 1000, and 2000 µg/g tumor) inside the tumor volume have been shown in Figure 4.

A similar trend as found for ^4^He can also be observed for variations of ^7^Li intensity, so that increasing the ^157^Gd concentration within the tumor volume decrements the intensity of the scored ^7^Li secondary particles. The main reason for continuous energy spectrum of ^7^Li is the multi‐energy nature of incident neutrons. As the neutron energy increments, lithium ions with higher energies would be released. However, neutron absorption cross‐section of ^10^B reduces with increasing the neutron energy (as illustrated in Figure 2). Therefore, it can be expected that the intensity of released ^7^Li particles continuously decreases when the lithium energy increments.

The observed sharp peaks in Figure 3 and Figure 4 (around 1.5 and 1.7 MeV for ^4^He and 0.8 and 1.09 MeV for ^7^Li) are relevant to the Boron neutron capture process, as shown in Equation (1). In this process, two nuclei with total energy above 2 MeV are generated which are recoiled in a back‐to‐back manner with equal momentums. Considering the energy and energy conservation laws, the obtained energies during the neutron capture process by Boron agents (2.31 and 2.79 MeV) are finally shared between ^4^He and ^7^Li based on their mass ratio.^13^

**FIGURE 5:**
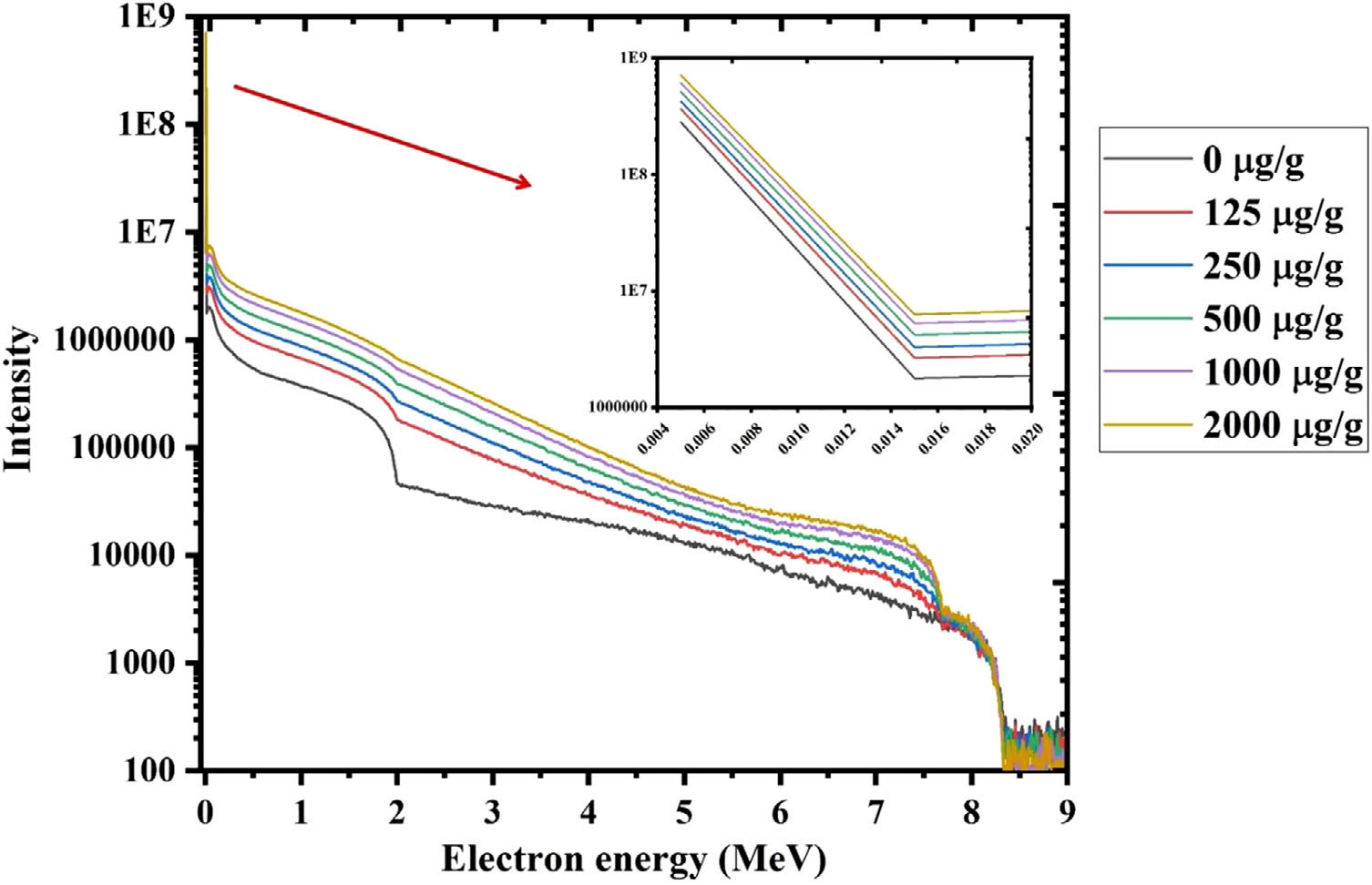
Scored energy spectra of total released secondary electrons during the neutron capture process by different concentrations of ^157^Gd during the combined Gd/BNCT technique.

The scored secondary electron spectra inside the tumor volume during the combined Gd/BNCT technique at different ^157^Gd concentrations have been shown in Figure 5.

As indicated in Figure 5, increasing the ^157^Gd concentrations inside the tumor region can increment the intensity of the secondary electron energy spectra due to the increment of neutron capture interactions at higher ^157^Gd concentrations.

**FIGURE 6:**
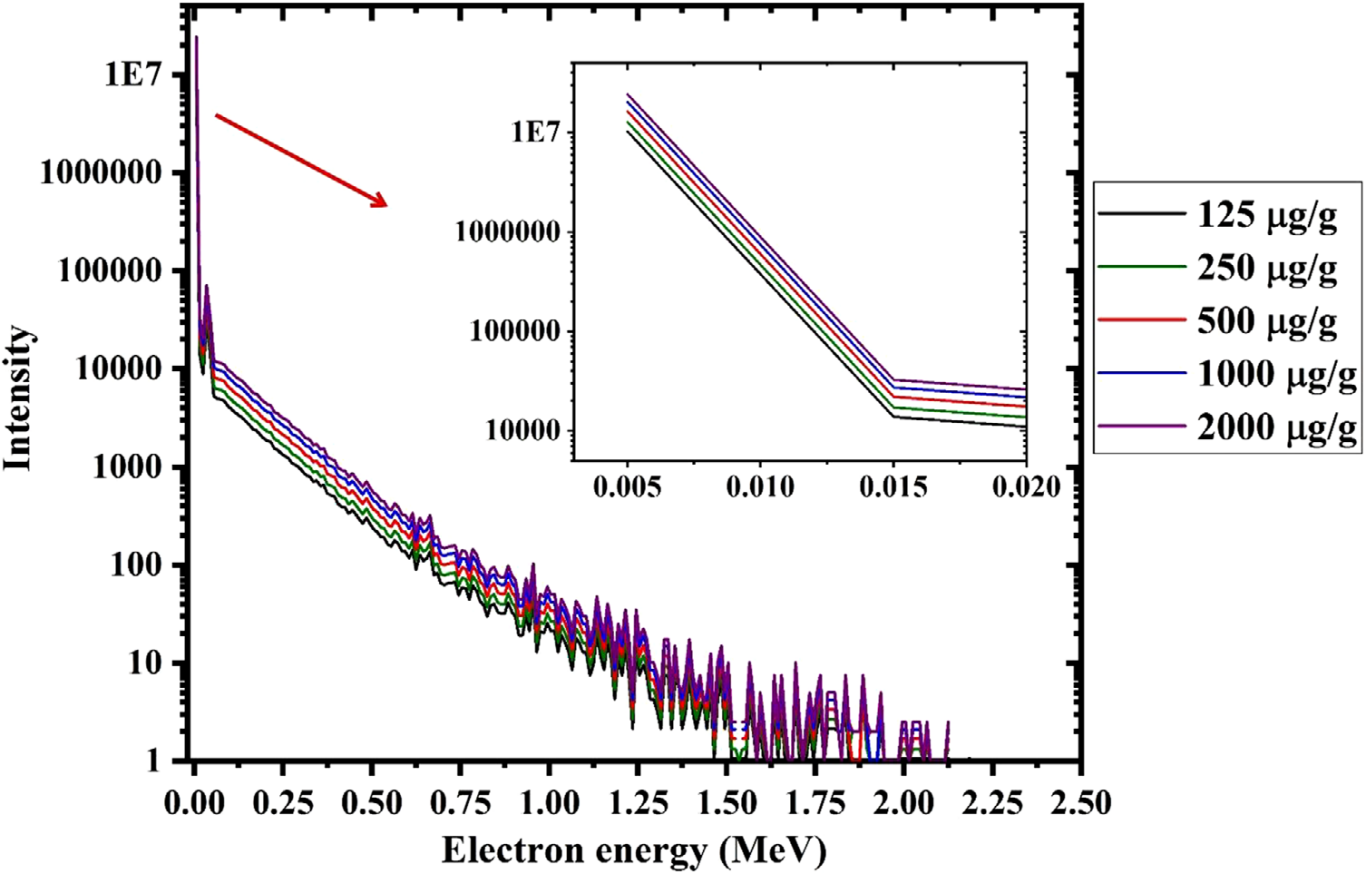
Scored energy spectra of released Auger electrons during the neutron capture process by different concentrations ^157^Gd during combined Gd/BNCT technique.

The contribution of Auger electrons in total electron spectra (as shown in Figure 5) has been illustrated in Figure 6. It should be noted that these Auger electrons have the most important role in determining the RBE value for ^157^Gd‐based neutron therapy technique.

As it can be seen in Figure 6, when the ^157^Gd concentration increments from 125 to 2000 µg/g inside the tumor region, the contributions of the low energy Auger electrons increment as well. This finding can be linked to the higher interaction rate of thermal neutrons with ^157^Gd when gadolinium concentration increments within the tumor region. Owing to the fact that these released Auger electrons cause a high LET value and cell killing probability, the radiation efficacy may be improved especially when the ^157^Gd accumulation would be nearby the DNA molecule.^16^, ^17^, ^38^ Since most of released energy during the neutron capture by ^157^Gd reaches to the gamma rays, if the Gadolinium is positioned outside the tumor cell, only gamma rays can contribute to the tumor cell killing.^38^

### 3.2 Dose calculations

**FIGURE 7:**
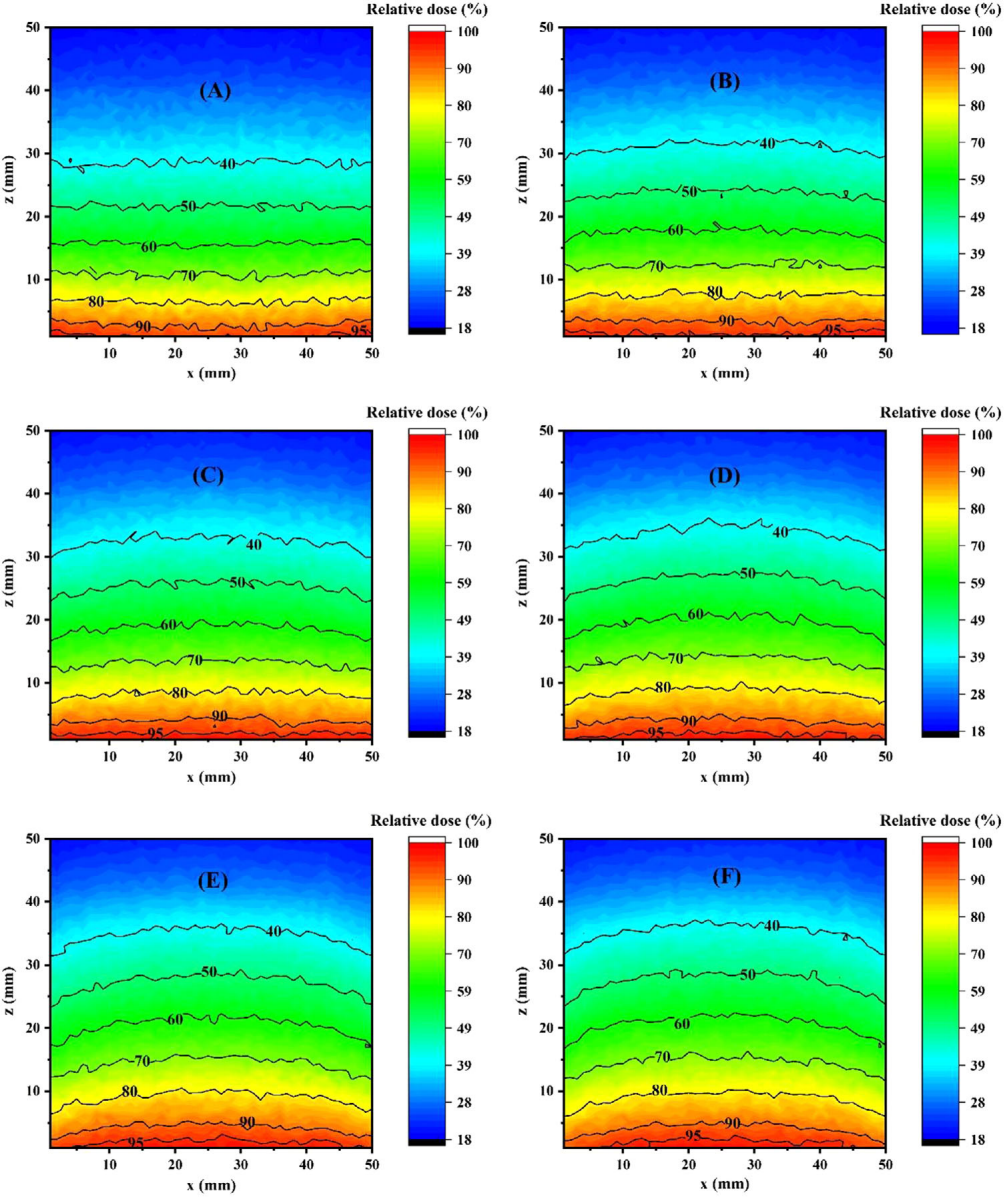
2D dose distribution inside the tumor volume as the function of ^157^Gd concentration; (A) 0 µg/g, (B) 125 µg/g, (C) 250 µg/g, (D) 500 µg/g, (E) 1000 µg/g, and (F) 2000 µg/g within the tumor region.

Figure 7 shows the simulated two dimensional (2D) dose distribution inside the tumor region for combined Gd/BNCT technique at different concentrations of ^157^Gd.

It is worth to note that all reactions which can affect the absorbed dose inside the tumor region were considered in the present study. When thermal neutrons penetrate to the tissues, due to the reactions of biological tissue compositions (^14^N(n,p)^14^C and ^1^H(n,ɣ)^2^H), the absorbed dose inside the target can be affected. Nevertheless, secondary particles released by neutron capture reaction are the main contributors to the received dose by the target.^23^

The illustrated results in Figure 7 showed a non‐uniform dose distribution is observed inside the tumor volume during both BNCT and combined Gd/BNCT techniques. This finding is expected because the neutron flux continuously reduces with penetrating to greater depths inside the tumor region. Ultimately, the absorbed dose decrements with increasing the depth inside the tumor volume. Nevertheless, when ^157^Gd is combined with ^10^B, more parts of the tumor region would be covered by the desirable dose values (red regions in Figure 7). These results can be attributed to the higher range of gamma rays and secondary electrons (products of ^157^Gd neutron capture) with respect to the heavy charged particles (as the products of ^10^B neutron capture) which can extend the depth of tumor coverage with higher dose levels. This issue would be more dominant when the ^157^Gd concentrations inside the tumor region increments.

### 3.3 RBE assessments

**FIGURE 8:**
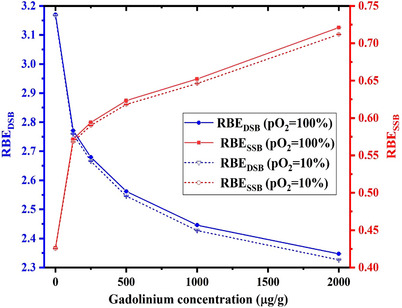
Calculated fluence‐averaged RBE values (RBE_SSB_ and RBE_DSB_) relevant to the combination of BNCT and GdNCT techniques for various ^157^Gd concentrations from 0 to 2000 µg/g inside the tumor volume as well as different cell oxygen levels.

The calculated fluence‐averaged RBE values (both RBE_SSB_ and RBE_DSB_) relevant to the various ^157^Gd concentrations inside the tumor volume and different cell oxygen levels have been illustrated in Figure 8.

As indicated in Figure 8, fluence‐averaged RBE_SSB_ values increment with an increase in ^157^Gd concentration. In this regard, fluence‐averaged RBE_SSB_ value increases by about 1.7 times when ^157^Gd concentration increments from 0 to 2000 µg/g for both considered cell oxygen levels (pO_2 _= 10% and 100%) in this study. RBE_SSB_ has a direct relationship with the high energy electrons which may induce single‐strand breaks with higher probability.^39^ On the other hand, the intensity of high energy secondary electrons which are released following the Gd‐based neutron capture increases at higher ^157^Gd concentrations, as shown in Figure 5. Therefore, it can be expected that the fluence‐averaged RBE_SSB_ values increase when Gadolinium uptake inside the tumor increments.

As shown in Figure 8, fluence‐averaged RBE_DSB_ values decrement when the ^157^Gd concentration increases from 0 to 2000 µg/g. Accordingly, a reduction of about 26% can be observed when ^157^Gd concentrations increment inside the tumor for both studied cell oxygen levels (pO_2 _= 10% and 100%). When the Gadolinium concentration increases inside the tumor region, the neutron flux is further reduced due to the increased number of neutron capture reactions by the ^157^Gd. This issue can consequently reduce the number of Boron neutron capture reactions and ultimately lead to the decrement of heavy charged particles (^4^He and ^7^Li) which are released following the Boron neutron capture reaction. On the other hand, these heavy charged particles are high‐LET ones which can induce the double strand breaks with a much higher probability than the products of Gadolinium neutron capture reaction. Regarding to the fact that the intensity of these heavy charged particles decrements with increasing the ^157^Gd concentration inside the tumor volume (as can be explicitly observed in Figure 3 and Figure 4), it can be expected that the RBE_DSB_ value reduces at higher ^157^Gd concentrations.

As illustrated in Figure 8, the obtained fluence‐averaged RBE_DSB_ values during the combined Gd/BNCT are lower than those reported for the sole BNCT which can be attributed to the reduction of heavy charged particle intensities, as discussed earlier. Furthermore, from the results of Figure 8, when the cell oxygen level varies from 10% to 100%, the fluence‐averaged RBE_SSB_ and RBE_DSB_ values increment with the maximum values of about 1.27% and 1% when the Gadolinium concentration is about 2000 µg/g inside the tumor region. Increasing the fluence‐averaged RBE_SSB_ and RBE_DSB_ values with the increment of ^157^Gd can be linked to the fact that the indirect induced damages to the DNA molecule by chemical products (hydroxyl radicals) may increase at higher levels of cell oxygen levels.

High LET particles such as alpha and lithium ions are produced during the BNCT which can lead to local energy deposition and improved RBE value. Nevertheless, very short range of these secondary ions (alpha, and lithium) and their local energy deposition nature may cause dose non‐uniformity inside the target volume. Although the released secondary particles during GdNCT have lower LET values with respect to BNCT, their long ranges inside the tissue can improve the dose distribution inside the target volume. However, due to the lower LET values, decreased RBE values are expected using GdNCT. Hence, combined GdNCT and BNCT for tumor irradiation can be beneficiary viewpoint to the dose distribution inside the target volume and can be harmful regarding the biological effectiveness of the delivered treatment to the patient. Finally, appropriateness or inappropriateness of the combined Gd/BNCT technique would be dependent on the followed priorities by the treatment team. In other words, if dose uniformity has priority, Gd/BNCT would be appropriate for treatment and if the clinical RBE is preferable, this combined technique can be considered as inappropriate method for radiotherapy.

Although the dose‐weighted RBE is a more appropriate metric for biological evaluation, the fluence‐averaged RBE (especially when different particle types contribute to the received dose by the target volume) can be also valuable for evaluating the effect of Gadolinium concentration during the tumor irradiation with combined Gd/BNCT technique on the absorbed dose distribution and RBE values. Nevertheless, the dose‐weighted RBE values may be different from fluence‐weighted RBE one, especially in the case of high LET particles such as alpha and helium ions. Accordingly, further considerations are needed to obtain the dose‐weighted RBE values which can be considered in our future studies.

The RBE_DSB_ values corresponding to the produced secondary particles (^7^Li and ^4^He) in BNCT have been estimated by Qi et al.^40^ In this study, the obtained RBE_DSB_ of alpha particles and ^7^Li nucleus were 3.27 and 3.44, respectively. Besides, for ^157^Gd the RBE_DSB_ values of about 1.5 outside of a considered cylindrical DNA molecule with 3 nm radius have been quantified by Cerullo et al.^41^

## 4 CONCLUSION

The DNA‐damage fluence‐averaged RBE values and the dose distribution during the NCT of brain tumor with ^10^B and ^157^Gd combination were assessed through a hybrid MC simulation approach in the current study. From the obtained results, it can be concluded that the depth dose distribution inside the tumor volume improves when the ^157^Gd agent is added to ^10^B for brain tumor irradiation, but in the expense of decreased fluence‐averaged RBE_DSB_ value which has an important role in clinical efficacy of this treatment modality. Nevertheless, to further improve the clinical efficacy of brain tumor treatment and reduce the relevant side‐effects towards normal tissues through the combined Gd/BNCT technique, the concentration ratio of ^10^B to ^157^Gd should be optimized through future investigations.

## AUTHOR CONTRIBUTIONS

Reza Shamsabadi: Methodology, validation, formal analysis, investigation, data curation, investigation, writing—original draft, writing‐ review & editing. Hamid Reza Baghani: Conceptualization, methodology, formal analysis, writing‐ review & editing, visualization, project administration, supervision.

## CONFLICT OF INTEREST STATEMENT

The authors declare no conflicts of interest.

## Data Availability

Data will be available upon reasonable request from the corresponding author.
